# Health and work disability outcomes in parents of patients with schizophrenia associated with antipsychotic exposure by the offspring

**DOI:** 10.1038/s41598-020-58078-4

**Published:** 2020-01-27

**Authors:** Heidi Taipale, Syed Rahman, Antti Tanskanen, Juha Mehtälä, Fabian Hoti, Erik Jedenius, Dana Enkusson, Amy Leval, Jan Sermon, Jari Tiihonen, Ellenor Mittendorfer-Rutz

**Affiliations:** 1 Karolinska Institutet, Department of Clinical Neuroscience, Stockholm, Sweden; 2 University of Eastern Finland, School of Pharmacy, Kuopio, Finland; 3 University of Eastern Finland, Department of Forensic Psychiatry, Niuvanniemi Hospital, Kuopio, Finland; 4 EPID Research Oy, Espoo, Finland; 5 Janssen Cilag, Solna, Sweden; 6 Janssen Cilag, Beerse, Belgium; 7 Center for Psychiatry Research, Stockholm City Council, Stockholm, Sweden

**Keywords:** Epidemiology, Epidemiology

## Abstract

This study aimed to identify if antipsychotic exposure in offspring is associated with psychiatric and non-psychiatric healthcare service use and work disability of their parents. This Swedish population-based cohort study was based on data comprising 10,883 individuals with schizophrenia, who had at least one identifiable parent in the nationwide registers, and their parents (N = 18,215). The register-based follow-up during 2006–2013 considered the level of antipsychotic exposure and persistence of use of the offspring, further categorized into first (FG) and second generation (SG) antipsychotics, and orals versus long-acting injections (LAIs). The main outcome measure was parental psychiatric healthcare service use, secondary outcomes were non-psychiatric healthcare use and long-term sickness absence. SG-LAI use was associated with a decreased risk (relative risks [RR] 0.81-0.85) of parental psychiatric healthcare use compared with not using SG-LAI, whereas oral antipsychotics were associated with an increased risk (RRs 1.10–1.29). Both FG- and SG-LAI use by the offspring were associated with a lower risk of long-term sickness absence (range of odds ratios 0.34–0.47) for the parents, compared with non-use of these drugs. The choice of antipsychotic treatment for the offspring may have an impact on work disability and healthcare service use of their parents.

## Introduction

Schizophrenia is a severe psychiatric disorder, affecting more than 21 million people globally^1^. The lifetime prevalence of schizophrenia has been traditionally estimated to be around 1%^2^, although more recent estimates provided are 0.4–0.7%^3,4^. The core features of schizophrenia are positive and negative symptoms, and cognitive impairment^5^. These often lead to chronic problems and disabilities^6,7^, and approximately 80% of persons with schizophrenia receive disability pensions and very few are employed^8^.

Non-adherence to antipsychotics among persons with schizophrenia is a major issue as about every third patient is estimated to be non-adherent with the antipsychotic treatment^9^. High rates of treatment discontinuation have been reported for both chronic patients^10,11^ and first-episode patients^12,13^, ranging from 32 to 84%, with somewhat varying follow-up times between the studies. The choice of antipsychotic drugs also impacts discontinuation rates as some studies report that first-generation (FG) antipsychotics are more likely to be discontinued than second-generation (SG) antipsychotics^14^. Furthermore, long-acting injectable (LAI) antipsychotics are less likely discontinued than oral antipsychotics^15,16^, even when LAI are compared directly with the same drug in oral formulation^17^.

Non-adherence to antipsychotic treatment has been associated with relapses, poorer life satisfaction, substance- and alcohol abuse and even suicides^18,19^. Besides inducing hazards for the patient, these outcomes may also stress parents or other caregivers significantly^20^. The burden on parents of the patient with the disorder is often presented as informal caregiving, i.e. based on a pre-existing personal relationship and without compensation for time or money spent, and it can be categorized into objective and subjective burden^21^. Objective burden includes time and money spent on care, whereas subjective burden includes an individual’s own perception of the burden. Both objective and subjective caregiver burden may lead to working disability including sickness absence and disability pension^22^. Schizophrenia also impacts the physical, psychological, emotional, social and financial life of caregivers for patients with schizophrenia^23^.

Subjective family burden of schizophrenia is higher when the patient has lower levels of functioning^21,24^, apparent psychotic symptoms or abnormal behaviour^25–27^, negative symptoms^28^, and when providing frequent caregiving assistance, monitoring medication and having limited social support^29^. In addition, risk of medication discontinuation and concerns about medication effectiveness are two significant indicators of caregivers’ distress^29^. Poor adherence to antipsychotic treatment has been associated with caregiver burden and experience of anxiety by family caregivers^30^.

In our previous study, we found that parents of patients with schizophrenia have a considerably higher risk of psychiatric health care use than parents of patients with multiple sclerosis (MS), rheumatoid arthritis, epilepsy or healthy controls^31^. Our recent observational studies have shown that LAIs were associated with lower risk of psychiatric re-hospitalizations compared with oral antipsychotics^32,33^ although this is contrary to the results of randomized trials^34,35^. Due to the associated lower risk of relapses and non-adherence, LAIs could impact the health and wellbeing of parents of patients with schizophrenia as re-emergence of psychotic symptoms and non-adherence are also drivers of caregiver burden^25–27,29,30^. However, there has been no research to date investigating the potential effects of antipsychotic treatment on caregiver burden, particularly on the parents. This study aimed to identify how antipsychotic exposure in the offspring with schizophrenia may affect healthcare resource use and work disability of their parents. A further aim was to investigate the association between persistence with antipsychotic medications among the offspring and risk of parental health and work disability outcomes.

Despite extensive research on the causative

## Methods and Materials

### Study design and population

This is a Swedish population-based cohort study based on individual level data collected from different nationwide registers. All residents in Sweden have a unique personal identifier, based on which all information on schizophrenia patients and their parents were linked at individual level. Initially, the patients were detected through national patient registers and thereafter parents through the Multi-generation register. The latter provided also information on the number of children of the parents. Additional information was derived from the following nationwide registers:

1. Longitudinal integration database for health insurance and labour market studies (LISA) held by Statistics Sweden, provided sociodemographic information on sex, age, educational level, area of residence and family situation.
2. National patient registers, maintained by the National Board of Health and Welfare (NBHW), containing information regarding the diagnoses and dates for in- or specialized outpatient care.
3. Prescribed drug register (from NBHW) provided data on type of medication, dispensing date, defined daily dose (DDD) and the Anatomical Therapeutic Chemical Classification System (ATC) code.
4. Cause of death register, held by NBHW, including dates and causes of death.
5. Micro-data for analyses of the social insurance (MiDAS) register, from the National Social Insurance Agency, containing information on date and diagnoses of sickness-absence (SA) and disability pension (DP).

### Offspring with schizophrenia

All individuals aged between 16–45 years and living in Sweden at the cohort entry date (CED), with a diagnosis of schizophrenia between July 01, 2006 and December 31, 2013, and with at least one identifiable parent were identified and included in the cohort (N = 10 883). Patients with schizophrenia were identified by using the International Classification of Diseases version 10 (ICD-10) codes F20 or F25 as a main diagnosis in the following instances: either discharged from inpatient care, or visit at specialized outpatient care, or sickness absence (SA), or disability pension (DP). Diagnoses were given by physicians after clinical assessment. The cohort entry date (CED) was set as the earliest of any of these events since July 01, 2006.

### Parents of schizophrenia cases

For 10 883 patients with schizophrenia (including both prevalent and incident, i.e. newly diagnosed cases), at least one parent was identified from the Multigeneration register (parent also had to be alive at time when follow-up started for the patient). The CED of the offspring was set as beginning of the follow-up for their parents. Originally, 19,065 parents were identified and after exclusions due to duplicates (i.e. parent had multiple children with schizophrenia) and due to parental mortality and emigration from Sweden during the CED year, 18,215 parents with an offspring with schizophrenia were included in the study.

### Exposure measures

Annual antipsychotic exposure was defined for patients with schizophrenia. Antipsychotics were defined according to the Anatomic Chemical Therapeutic (ATC) classification system, as N05A, excluding lithium. Antipsychotics were further categorized into first-generation (FG) and second-generation (SG) medications, and into oral and long-acting injection (LAI) formulations, forming subgroups of FG-oral, SG-oral, FG-LAI, and SG-LAI (Supplementary Table 1).

Treatment duration was modelled from drug dispensing with the PRE2DUP method^36^. The method is based on calculation of sliding averages of the daily dose and it takes individual variation into account, as well as hospital days (time when medication use is not recorded in the Prescribed Drug register) and stockpiling of drugs. For this study, a switch between medications was defined as a concomitant use of ≤30 days and polytherapy as a concomitant use of >30 days. A switch from a previous antipsychotic drug to a new drug was defined as taking place at the initiation of the new drug (previous drug was then discontinued at this point). Each antipsychotic medication was modelled separately and considered when defining “any antipsychotic exposure”. For categorizations such as “FG-oral exposure”, overlapping exposure periods of this category were combined to retrieve the time on any FG-oral medication. This was compared with time not using this specific medication, i.e. non-use of FG-oral medication (including use of all other categories and no use of antipsychotics).

The annual antipsychotic and polypharmacy exposures of the schizophrenia patients were defined according to the cumulative number of exposed days during a calendar year, for each study year (2006–2013). To account for different follow-up times, the cumulative number of exposure days was divided by the number of follow-up days, i.e. excluding hospital days and censoring to mortality. Annual exposures were categorized as ‘no exposure’ if there was not on-going antipsychotic use at all during the year; ‘low exposure’ assigned to those who had on-going exposure during the calendar year that lasted at most 50% of days within that year, excluding hospital days; and ‘moderate to high exposure’ (short as “moderate exposure”) which was assigned to those who had more than 50% days of on-going exposure during the calendar year, excluding hospital days. The categorization was made for each study year and thus, a patient may change exposure group during the study.

Treatment persistence with antipsychotic medication was calculated annually and categorized as ‘no use’ if there was not any on-going antipsychotic use during the year, ‘low persistence’ assigned to those who had longest antipsychotic treatment period during the calendar year that lasted at most 50% of the days of a calendar year excluding hospital days, ‘medium persistence’ was assigned to those who had more than 50% and less than 100%, and ‘high persistence’ was assigned to those who had 100%.

### Outcome measures

Outcomes were defined for parents of patients with schizophrenia. Healthcare resource use was calculated as the number of inpatient and specialized outpatient care visits due to mental and behavioural disorders, named as “psychiatric healthcare use” (ICD-10 codes: F00-F99) and non-psychiatric disorders (including diabetes mellitus type 2; diseases of the circulatory system; diseases of esophagus, stomach and duodenum; liver disease and dorsalgia: ICD-10 codes: E11-E14, I00-I99, K20-K31, K70-K77, M54, respectively). Information on the number of visits was used as a continuous measure. Work disability was measured by considering sickness absence (SA) for more than 90 annual gross days which is a common limit used in work disability research. It was assessed annually during the follow-up and dichotomized.

### Covariates

For patients, information on sex, age, severity level and number of different antipsychotics used in the past were derived. The number of different antipsychotics used in the past was assessed as count of different antipsychotics used prior to start of each calendar year and yearly updated, categorized as 1, 2–3, >3. For their parents, sex, age, calendar year, education, region of residence, medication used within the calendar year, and family situation were obtained. Covariates were categorised as shown in Table 1. Medication use included use of the following medications (ATC codes): drugs for acid-related disorders (A02), analgesics (M01-M05, N02), cardiovascular drugs (C01-C10), drugs for liver disease (J05AF), antipsychotics (N05A), antidepressants (N06A) and drugs for anxiety and sleep disturbances (N05B-C). A further covariate in the analyses was severity level of patients with schizophrenia, which was based on the number of psychiatric hospitalizations (in hospital care at least overnight) due to psychosis during the follow-up (ICD-10 F20-F29). The patients were defined as belonging to the 1st quartile (25% of patients with the lowest number of visits) as ‘least severe’, those in the 2nd – 3rd quartiles (50% of patients) as ‘moderately severe’ and those in the 4th quartile (25% of patients with most visits) as ‘most severe’. Age, sex and severity level were assessed at CED and other variables were constantly updated in the models (i.e. time-dependent variables re-assessed at the beginning of each follow-up year). Education, region of residence and family situation variables included missing data which formed a category in the analyses.

**Table 1:** Baseline characteristics of the patients with schizophrenia (N = 10 883) and their parents (N = 18215) included in this study.

Characteristics	Schizophrenia patients n (%)
**Sex**, males	6864 (63.1)
**Age at cohort entry (years)**^**a**^	
16–24	1138 (10.5)
25–34	3693 (33.9)
**≥**35	6052 (55.6)
Median age (IQR)	36 (29–41)
**Severity level of schizophrenia**^**a**^	
Most severe patients	1237 (11.4)
Moderately severe patients	2469 (22.7)
Least severe patients	6930 (62.9)
Patients with no follow-up	247 (2.3)
**Number of different antipsychotics used during the year of cohort entry**^**a**^	
0	1114 (10.2)
1	5657 (52.0)
2	3204 (29.4)
3	777 (7.1)
>3	131 (1.2)
**Characteristics**	**Parents of schizophrenia patients n (%)**
**Sex**, males	8186 (44.9)
**Age at start of follow-up (years)**^**a**^	
35–44	215 (1.2)
45–54	2708 (14.9)
55–64	7138 (39.2)
≥65	8154 (44.7)
Median age (IQR)	63 (57–69)
**Year of start of follow-up**	
2007	10 255 (56.3)
2008	3036 (16.7)
2009	1932 (10.6)
2010	1083 (5.9)
2011	886 (4.9)
2012	620 (3.4)
2013	403 (2.2)
**Area of residence**^**a**^	
Big cities	6496 (35.7)
Medium city	6422 (35.3)
Small cities	5291 (29.1)
Missing	6 (0.03)
**Educational level**^**a**^	
Low (≤10 years)	5372 (29.5)
Medium (10–12 years)	7537 (41.4)
High (>12 years)	4864 (26.7)
Missing	442 (2.4)
**Family situation**^**a**^	
Married, living without children	6208 (34.1)
Married, living with children	3844 (21.1)
Single, living without children	6304 (34.6)
Single, living with children	1853 (10.2)
Missing	6 (0.03)
**Outcome measures at baseline**^a^	
Specialised psychiatric healthcare	1067 (5.9)
Specialised non-psychiatric healthcare	1631 (9.0)
Sickness absence >90 days	791 (4.3)

^a^Measured at cohort entry (CED).

IQR = interquartile range.

### Statistical analyses

Analyses were performed on the population of parents with up to seven years of follow-up. Parents were grouped according to the antipsychotic exposure of their offspring (FG-Oral, FG-LAI, SG-Oral, and SG-LAI; and on substance level). To ascertain that exposure of the offspring took place before the outcome of the parent, exposure of the offspring during the previous year was the independent explanatory variable of interest. Main analyses considered annual antipsychotic exposure and additional analyses were conducted with treatment persistence as an exposure. Logistic regression was utilized for ‘SA exceeding 90 days’, and Poisson regression for healthcare resource use outcomes. Follow-up started at the 1^st^ of January of the year following CED and continued until the death of the caregiver or the patient, or the end of the study period, whichever occurred first. The calendar year of CED was referred to as follow-up year 0, and follow-up year 1 was the first calendar year after year 0. The actual analyses considered the follow-up years 1–7. Parents with on-going DP at CED were excluded from analyses of SA. Analyses were adjusted for offspring/patient related variables: number of different antipsychotics used prior to the analyzed calendar year, age, sex, severity level; and parental related variables: medication use, calendar year, age, sex, family situation, and region of residence. The results are reported as rate ratios (RRs) and odds ratios (ORs) with 95% confidence intervals (CIs). Tables present the results with p < 0.01 as bolded, due to multiple exposure categories being tested and only these results were considered significant.

To investigate whether possible dependency between parents has impact on the results, sensitivity analysis was conducted for the main outcome measure (psychiatric healthcare use) by selecting only one parent for each person with schizophrenia.

### Ethical approval

The study is based on several Swedish national registers, which are linked for research purposes. All registers used for this study were anonymised and de-identified prior to analysis by Statistics Sweden, which was responsible for data linkage. Thus, researchers received de-identified data. In Sweden, ethical vetting is always required when using register data and performed by regional review boards, and the risk appraisal associated with the Law on Public Disclosure and Secrecy is performed by the register keepers. The ethical vetting was performed and approved by the Regional Ethical Review Board of Stockholm, Sweden, according to the Swedish Ethical Review Act and after that also by each of the different authorities/data keepers (Statistics Sweden, National Board of Health and Welfare and the National Social Insurance Agency) according to the Public Access to Information and Secrecy Act, the Personal Data Act, and the Administrative Procedure Act. Those ethical review boards can waive the requirement to consult the data subjects directly to obtain their informed consent. This is often the case if the research is supported by the ethical review board and the data have already been collected in some other context, e.g. routine data like insurance records, such as in this study.

## Results

The majority of the identified patients with schizophrenia were aged ≥35 (56%), with median age 36 years (interquartile range IQR 29–41) (Table 1). They were more likely to be men (63%), and more than two thirds (63%) of patients with schizophrenia were categorized as least severe, whereas only 11% were most severely ill.

The majority of the parents of patients with schizophrenia were female (55%), with a median age of 63 years (IQR 57–69) at the start of follow-up (Table 1). Parents most often living in big or medium sized cities (71%) and 69% were living without children (either single or married).

### Healthcare resource use

Moderate or low antipsychotic exposure for FG-oral and SG-oral by the patients with schizophrenia was associated with a slightly higher risk of psychiatric healthcare use among their parents (Range of RRs 1.10 to 1.29; p < 0.01, Table 2) compared with parents of patients who were not using these drug classes. Moderate exposure for SG-LAIs by the offspring was associated with a lower risk of psychiatric health care resource use in their parents (RR 0.81; CI: 0.75, 0.89; p < 0.01) compared with parents of patients who were not using SG-LAIs. These results remained similar in sensitivity analyses including only one parent for each person with schizophrenia (the results not shown).

**Table 2:** Antipsychotic (AP) use by the offspring with schizophrenia and associated adjusted rate ratios (RR) of parental healthcare use.

Antipsychotic exposure	Events	Person-years	RR^a^
**Psychiatric healthcare use**			
***Any AP use***			
- No	1318	7957	1
- Moderate	8764	64 086	0.97 (0.90, 1.04)
- Low	722	4888	0.92 (0.84, 1.02)
***FG-LAI use***			
- No	8859	63 962	1
- Moderate	1535	10361	1.05 (0.99, 1.11)
- Low	410	2608	0.94 (0.85, 1.04)
***FG-Oral use***			
- No	7618	58 428	1
- Moderate	2312	13512	**1**.**29 (1**.**22**, **1**.**36)**
- Low	874	4991	**1**.**10 (1**.**03**, **1**.**19)**
***SG-LAI use***			
- No	9971	70 760	1
- Moderate	622	4728	**0**.**81 (0**.**75**, **0**.**89)**
- Low	211	1444	0.85 (0.74, 0.98)
***SG-Oral use***			
- No	2828	20 630	1
- Moderate	6839	49760	**1**.**10 (1**.**05**, **1**.**16)**
- Low	1137	6540	**1**.**20 (1**.**11**, **1**.**29)**
**Non-psychiatric healthcare use**			
***Any AP use***			
- No	1694	7957	1
- Moderate	12840	64 086	**0**.**91 (0**.**85**, **0**.**97)**
- Low	885	4888	0.89 (0.82, 0.98)
***FG-LAI use***			
- No	12722	63 962	1
- Moderate	2203	10361	0.98 (0.93, 1.03)
- Low	494	2608	0.92 (0.84, 1.01)
***FG-Oral use***			
- No	11781	58 428	1
- Moderate	2708	13512	**0**.**92 (0**.**87**, **0**.**96)**
- Low	930	4991	0.92 (0.86, 0.99)
***SG-LAI use***			
- No	14088	70 760	1
- Moderate	1040	4728	1.08 (1.01, 1.16)
- Low	291	1444	1.06 (0.95, 1.20)
***SG-Oral use***			
- No	4352	20 630	1
- Moderate	9853	49760	0.97 (0.93, 1.02)
- Low	1214	6540	0.95 (0.89, 1.02)

Exposure categories comprise no antipsychotic (AP) use, low (<50% of days) and moderate (≥50% of days) use during the previous calendar year. For bolded ones, P < 0.01.

^a^Adjusted for offspring/patient related variables: number of different antipsychotics used prior to the analyzed calendar year, age, sex, severity level; and parental variables: medication use, calendar year, age, sex, family situation, region of residence. FG: first-generation antipsychotic; SG: second-generation antipsychotic; LAI: long-acting injectable antipsychotic.

Any antipsychotic exposure by the patients was associated with a somewhat lower risk of non-psychiatric healthcare use among their parents compared with no antipsychotic use (moderate use RR 0.91; CI:0.85, 0.97; p < 0.01) (Table 2). The group level analyses revealed that FG-oral use was associated with a decreased risk of non-psychiatric healthcare use among parents (moderate use- RR 0.92; CI: 0.87, 0.96; p < 0.01) compared with parents of patients not using FG-orals, whereas SG-LAIs were not associated with non-psychiatric healthcare use.

Of the antipsychotic drug substances, moderate exposure to oral flupentixol and levomepromazine of the offspring were associated with a 77% and 45% higher risk of psychiatric health care resource use among their parents compared to no use of these antipsychotics, respectively (p < 0.01, Table 3). Moderate exposure to paliperidone LAI and risperidone LAI by the patient were associated with lower risks of psychiatric health care resource use among their parents compared to no use of these antipsychotics (RR 0.56; CI: 0.36, 0.86; and RR 0.77; CI: 0.70, 0.84, respectively). Low exposure categories for certain drugs implied a higher or lower risk but not their moderate exposure category and thus, these were considered as inconclusive results.

**Table 3:** Exposure to specific antipsychotics (AP), the number of events, person-years (PY) by the offspring and associated adjusted rate ratios (RR) and 95% Confidence intervals (CI) of parental psychiatric healthcare use.

AP exposure	Events	PY	RR (95% CI)*
**FG-LAI**			
Haloperidol use			
No	10470	74 723	1
Moderate	252	1746	0.92 (0.81, 1.04)
Low	82	462	0.82 (0.66, 1.02)
**Perphenazine use**			
No	10148	72 602	1
Moderate	494	3255	1.00 (0.91, 1.10)
Low	162	1074	0.99 (0.85, 1.16)
**Zuclopenthixol use**			
No	9986	71 256	1
Moderate	642	4439	0.99 (0.91, 1.08)
Low	176	1237	0.85 (0.73, 0.99)
**FG-Oral**			
**Chlorprothixene use**			
No	10739	76 435	1
Moderate	23	191	0.80 (0.53, 1.21)
Low	42	305	0.77 (0.57, 1.05)
**Flupentixol use**			
No	10422	75 152	1
Moderate	290	1317	**1**.**77 (1**.**57**, **2**.**00)**
Low	92	462	**1**.**37 (1**.**12**, **1**.**69)**
**Haloperidol use**			
No	10153	72 851	1
Moderate	417	2866	0.92 (0.83, 1.02)
Low	234	1214	1.09 (0.96, 1.25)
**Levomepromazine use**			
No	9313	69 398	1
Moderate	1054	4927	**1**.**45 (1**.**35**, **1**.**56)**
Low	437	2606	1.01 (0.91, 1.11)
**Melperone use**			
No	10761	76 729	1
Moderate	21	117	1.48 (0.96, 2.28)
Low	22	85	1.51 (0.99, 2.29)
**Perphenazine use**			
No	10291	73 531	1
Moderate	316	2310	1.03 (0.92, 1.15)
Low	197	1090	1.04 (0.90, 1.20)
**Zuclopenthixol use**			
No	10224	72 671	1
Moderate	413	3042	0.95 (0.86, 1.05)
Low	167	1218	**0**.**77 (0**.**66**, **0**.**90)**
**SG-LAI**			
**Olanzapine use**			
No	10747	76 616	1
Moderate	41	168	1.24 (0.91, 1.69)
Low	16	148	0.58 (0.35, 0.95)
**Paliperidone use**			
No	10727	76 373	1
Moderate	21	236	**0**.**56 (0**.**36**, **0**.**86)**
Low	56	323	1.10 (0.84, 1.44)
**Risperidone use**			
No	10076	71 403	1
Moderate	561	4325	**0**.**77 (0**.**70**, **0**.**84)**
Low	167	1203	**0**.**81 (0**.**70**, **0**.**95)**
**SG-Oral**			
**Aripiprazole use**			
No	8553	62 539	1
Moderate	1526	10319	1.03 (0.97, 1.09)
Low	725	4074	**1**.**16 (1**.**07**, **1**.**25)**
**Clozapine use**			
No	8761	62 149	1
Moderate	1897	13825	1.00 (0.95, 1.06)
Low	146	957	0.98 (0.83, 1.16)
**Olanzapine use**			
No	7936	56 583	1
Moderate	2072	16026	0.96 (0.91, 1.01)
Low	796	4322	**1**.**13 (1**.**04**, **1**.**22)**
**Paliperidone use**			
No	10581	75 489	1
Moderate	95	727	0.89 (0.72, 1.09)
Low	128	715	1.16 (0.98, 1.39)
**Quetiapine use**			
No	9265	67 163	1
Moderate	1098	6991	1.01 (0.95, 1.08)
Low	441	2778	0.96 (0.87, 1.06)
**Risperidone use**			
No	9461	66 719	1
Moderate	962	7615	1.00 (0.93, 1.08)
Low	381	2598	0.99 (0.89, 1.11)
**Ziprasidone use**			
No	10508	74 511	1.00
Moderate	222	1852	0.86 (0.75, 0.99)
Low	74	569	**0**.**70 (0**.**56**, **0**.**88)**

Exposure categories no use, low (<50% of days) and moderate (≥50% of days) during previous calendar year. For bolded ones, P < 0.01.

*Adjusted for offspring/patient related variables: number of different antipsychotics used prior to the analyzed calendar year, age, sex, severity level; and parental variables: medication use, calendar year, age, sex, family situation, and region of residence. FG: first-generation antipsychotic; SG: second-generation antipsychotic; LAI: long-acting injectable antipsychotic.

### Long-term sickness absence

Use of FG-LAI and SG-LAI by the patient were associated with a lower risk for parental long-term SA compared with parents of patients not using these drug classes (moderate FG-LAI use OR 0.47; CI: 0.31, 0.69; moderate SG-LAI use OR 0.34; CI: 0.21, 0.55, p < 0.01) (Table 4). In contrast, moderate exposure to FG-oral increased such risk (OR 1.56; CI: 1.16, 2.10).

**Table 4:** Antipsychotic (AP) exposure, the number of events and person-years (PY) by the offspring and associated adjusted odds ratios (OR) and 95% Confidence interval (CI) of parental long-term sickness absence (>90 days).

AP exposure	Events	PY	OR (95% CI)
***FG-LAI use***			
- No	1166	52 303	1
- Moderate	136	8134	**0**.**47 (0**.**31**, **0**.**69)**
- Low	42	2011	**0**.**34 (0**.**19**, **0**.**60)**
***FG-Oral use***			
- No	1034	47 559	1
- Moderate	214	10969	**1**.**56 (1**.**16**, **2**.**10)**
- Low	96	3921	1.24 (0.89, 1.73)
***SG-LAI use***			
- No	1220	57 587	1
- Moderate	94	3755	**0**.**34 (0**.**21**, **0**.**55)**
- Low	30	1109	**0**.**47 (0**.**26**, **0**.**87)**
***SG-Oral use***			
- No	373	16 605	1
- Moderate	834	40649	0.80 (0.61, 1.04)
- Low	137	5196	1.35 (0.99, 1.84)

Exposure categories no antipsychotic, low (<50% of days) and moderate (≥50% of days) during previous calendar year. For bolded ones, P < 0.01.

*Adjusted for offspring/patient related variables: number of different antipsychotics used prior to the analyzed calendar year, age, sex, severity level; and parental variables: medication use, calendar year, age, sex, family situation, and region of residence. FG: first-generation antipsychotic; SG: second-generation antipsychotic; LAI: long-acting injectable antipsychotic.

### Persistence of use

Compared with medium persistence, high persistence of the offspring to antipsychotic medication was associated with a lower risk of parental psychiatric health care resource use (RR 0.90; CI: 0.83, 0.98) (Table 5). However, a similar result was observed with low persistence. In terms of non-psychiatric healthcare use, no use was associated with a somewhat increased risk of parental non-psychiatric health care use (RR 1.10; CI:1.02, 1.19), but other persistence categories were not. No association was found for SA (high persistence OR 1.15; CI: 0.78, 1.70)

**Table 5:** Persistence with antipsychotic (AP) use by the offspring and associated adjusted rate ratios (RR) with 95% Confidence interval (CI) of parental healthcare use and long-term sickness absence.

AP persistence	Events	PY	RR or OR (95% CI)^a^
**Psychiatric healthcare use (RR)**			
*-* No	1412	8678	0.92 (0.85, 1.00)
- Low	6896	50 370	0.89 (0.83, 0.94)
- Medium	1143	6982	1
- High	1353	10 902	0.90 (0.83, 0.98)
**Non-psychiatric healthcare use (RR)**			
*-* No	1771	8678	1.10 (1.02, 1.19)
- Low	10196	50 370	1.05 (0.99, 1.11)
- Medium	1286	6982	1
- High	2166	10 902	1.05 (0.98, 1.12)
**Sickness absence within the calendar year (>90 days) (OR)**			
*-* No	223	6860	1.38 (0.90, 2.12)
- Low	823	41 022	1.35 (0.99, 1.84)
- Medium	135	5612	1
- High	163	8955	1.15 (0.78, 1.70)

Persistence refers to coverage of the longest use period during the previous calendar year, with categories: no (as no AP use), low (≤50% of days), medium (51–99% of days) and high (100% of days).

^a^Adjusted for offspring/patient related variables: number of different antipsychotics used prior to the analyzed calendar year, age, sex, severity level; and parental variables: medication use, calendar year, age, sex, family situation, and region of residence.

## Discussion

### Main findings

To our knowledge, this is the first study assessing associations between antipsychotic use in the offspring with schizophrenia and parental health and work disability outcomes. FG- and SG-LAI use by the patient was associated with a lower risk of long-term sickness absence for their parents when compared with parents of patients who were not using LAIs. Oral antipsychotics were associated with an increased risk, but SG-LAIs with a decreased risk of parental psychiatric healthcare use compared with parents of patients who were not using SG-LAIs. In general, antipsychotic exposure by the patients with schizophrenia was associated with somewhat lower risk of non-psychiatric healthcare utilization among their parents compared to no use of antipsychotics. However, many of the differences found were rather small and only the association between SG-LAI use and parental outcomes was somewhat consistent across outcomes and analyses.

LAI use was associated with a lower risk of parental SA and SG-LAI use with a lower risk of parental psychiatric healthcare service use, whereas the same was not observed for oral antipsychotic use. These findings may be related to a better adherence to medication associated with LAI use compared with oral antipsychotics^15–17^. Better adherence may relieve parental concerns over discontinuation of medication use by their offspring and reduce the need for monitoring of medication taking behaviour^29^. Our previous study showed that parents of patients with schizophrenia in general have a higher risk of psychiatric health care use compared with parents of patients with MS, rheumatoid arthritis, epilepsy or healthy controls^31^.

The choice of antipsychotic drug substance and administration form (oral vs. LAI) in schizophrenia is not random and LAIs are often reserved for more severe or treatment resistant patients^37^. According to a previous study, LAI users had more psychiatric hospitalizations in their history, alcohol and illicit substance use, were more likely to have been arrested and had had more often psychotic symptoms, compared with oral antipsychotic users^37^. Against this background, findings on better parental outcomes associated with LAI use is somewhat surprising. However, besides better adherence, LAI use is associated with a lower risk of relapses^32,33^ which may lower parental distress and worrying over the wellbeing of their offspring. Previous studies have shown that psychotic symptoms in the patients with schizophrenia are associated with higher family burden^25^ and therefore, more consistent antipsychotic use by patients may explain our findings on a lower risk of healthcare use of the parents. The study by Lerner *et al*. (2018) indicated that parents are also concerned about the effectiveness of antipsychotic medication in controlling psychotic symptoms. For this reason, a lowered risk of psychiatric hospitalizations for the patients (as a marker for re-emergence of psychosis) may relieve these concerns.

Our findings on the protective effect of moderate exposure to SG-LAI in patients regarding psychiatric specialized healthcare use in parents can be discussed in the light of our previous study on comparative effectiveness of antipsychotics in preventing psychiatric re-hospitalizations^32^. SG-LAIs were found to be associated with a lower risk of psychiatric re-hospitalization in the patients in the previous observational study. This might in turn have an effect on the parents. Of specific LAIs, risperidone LAI and paliperidone LAI use by the patient were associated with lower parental psychiatric health care use.

Any antipsychotic exposure for the offspring with schizophrenia was associated with a lower risk of parental non-psychiatric healthcare use, but group-wise analyses were somewhat less consistent. FG-oral antipsychotics were associated with a somewhat lower risk of non-psychiatric healthcare use. SG-LAI use showed a tendency towards higher non-psychiatric healthcare use of the parents. Due to lack of consistency between drug classes and exposure categories no definitive conclusions can be made.

Both FG- and SG-LAI use by the offspring was associated with a 50–60 percent lower risk of parental long-term sickness absence. This may indicate that LAI use by the patient with schizophrenia may reduce caregiver burden as previous studies have linked caregiver burden with increased likelihood of sickness absence^22^. The mechanisms would likely be related to better adherence to medication, less frequent psychotic symptoms and reduced need for assistance provided by the parent^25–27,29^. However, using this type of non-interventional study design, causality cannot be assessed, and future studies should be conducted on whether LAI use in general or switch from oral to LAI by the patient would decrease parental caregiver burden.

### Strengths and limitations

The strengths of this study include the nationwide coverage of patients with schizophrenia and their parents, with long-term follow-up with register-based data. In this study, we focused on persons with schizophrenia aged 16–45 years to maximise the possibility that their parents are still alive.

A major strength of this study is also the identification of person-level drug exposure and modelling drug dispensing data into longitudinal drug exposure periods with the validated PRE2DUP method^36,38^. In this study, we utilized two measures of antipsychotic exposure. Antipsychotic exposure time during previous year was the main exposure measure. In secondary analyses on persistence of antipsychotic use, the longest continuous drug exposure period during the previous year was assessed. The first measure allows breaks in drug use whereas high persistence requires continuous and regular use throughout the year. Both represent patterns of cumulative use in the past so that there is time for parental outcomes to develop. In addition, assessment of exposure of the previous year in the patients prior to the year of the measurement of parental outcomes ensured that exposure happened before the outcome event. However, as parental outcomes were assessed in yearly time windows it is possible that utilized outcome measures may miss some acute developments in parental health.

Limitations include the lack of some important covariates. For example, patients’ satisfaction with their medication or parent’s satisfaction with their offspring’s medication, perceived quality of life and life style factors could not be assessed. The severity level of patients with schizophrenia was measured as the number of psychiatric hospitalization (as a proxy for relapses) during the follow-up. However, it is possible that temporal changes in the severity are not accounted for. The measurement of sickness absence builds on information from the Social insurance agency. This results in the fact that information on the first 14 days of sickness absence of employees is not included. Moreover, the nature of the association between offsprings’ antipsychotic exposure and parents’ health development and work disability could not be assessed, as we don’t have the information if parents actually were caregivers for their children.

## Conclusions

We found LAI use by the offspring to be associated with a lower risk of parental sickness absence and SG-LAI use with lower parental psychiatric healthcare service use. Choice of antipsychotic for the offspring may have an association with work disability and health care service use of their parents.

## Supplementary information

Supplementary material.
