# Supplementary material for: Health and work disability outcomes in parents of patients with schizophrenia associated with antipsychotic exposure by the offspring
Source: Sci Rep. 2020 Jan 27;10:1219. doi: 10.1038/s41598-020-58078-4 (PMC6985214; doi:10.1038/s41598-020-58078-4)
Supplement: Supplementary file 1 — Supplementary material. [file 41598_2020_58078_MOESM1_ESM.pdf]

Supplementary material

Health and work disability outcomes in parents of patients with schizophrenia associated with antipsychotic exposure by the offspring

Heidi Taipale, Syed Rahman, Antti Tanskanen, Juha Mehtälä, Fabian Hoti, Erik Jedenius,

Dana Enkusson, Amy Leval, Jan Sermon, Jari Tiihonen, Ellenor Mittendorfer-Rutz

Supplementary Table 1. Antipsychotics used by the study population and the corresponding ATC codes.

**SG-Oral**	**ATC code**
Risperidone	N05AX08
Olanzapine	N05AH03
Paliperidone	N05AX13
Ziprasidone	N05AE04
Aripiprazole	N05AX12
Sertindole	N05AE03
Quetiapine	N05AH04
Clozapine	N05AH02
**SG-LAI**	
Risperidone	N05AX08
Olanzapine	N05AH03
Paliperidone	N05AX13
Aripiprazole	N05AX12
**FG-Oral**	
Fluphenazine	N05AB02
Perphenazine	N05AB03
Haloperidol	N05AD01
Flupentixol	N05AF01
Zuclopenthixol	N05AF05
Levomepromazine	N05AA02
Melperone	N05AD03
Chlorprothixene	N05AF03
**FG-LAI**	
Haloperidol	N05AD01
Fluphenazine	N05AB02
Perphenazine	N05AB03
Flupentixol	N05AF01
Zuclopenthixol	N05AF05

FG: first-generation antipsychotic; SG: second-generation antipsychotic; LAI: long-acting injectable antipsychotic.
